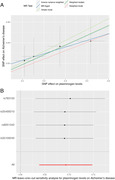# Genetically determined higher circulating plasminogen levels contribute to an increased risk of Alzheimer’s disease

**DOI:** 10.1002/alz.088482

**Published:** 2025-01-09

**Authors:** Xingzhi Guo, Rui Li

**Affiliations:** ^1^ Shaanxi Provincial People’s Hospital, Xi'an, Shaanxi China

## Abstract

**Background:**

Alzheimer's disease (AD) is the most common form of dementia, characterized by cognitive impairment and memory loss. Previous studies have demonstrated that plasminogen, a key molecule in the fibrinolytic system, is implicated in the pathophysiology of AD. However, it is yet unknown whether the relationship between blood plasminogen and AD is causal.

**Methods:**

Leveraging instrumental variables (IV) from genome‐wide association studies (GWAS), we performed a two‐sample Mendelian randomization (MR) analysis to investigate the causal relationship between plasminogen levels and AD risk. Summary‐level data on blood plasminogen levels was from a GWAS on human blood plasma proteome, and summary statistics on AD were from the International Genomics of Alzheimer's Project (IGAP, N=63,926). Inverse variance weighted (IVW) was used as the primary approach to calculate the estimate, which was further validated using the following sensitivity analyses, including MR‐Egger, Mendelian Randomization Pleiotropy RESidual Sum and Outlier (MR‐PRESSO), weighted median, simple mode, and weighted mode methods. Additionally, tests for pleiotropy and heterogeneity were conducted to further assess the stability of the MR estimates.

**Results:**

Using the IVW approach, our findings revealed a significant association between higher genetically determined plasminogen levels and an increased risk of AD (odds ratio [OR] = 1.10, 95%CI = 1.04‐1.16, P =0.008), which was further confirmed in sensitivity analyses (Figure 1A). The leave‐one‐out analysis plot revealed that the causal estimate between plasminogen levels and AD was not driven by any single instrumental variable (Figure 1B). The findings from the MR‐Egger intercept test and Cochran's Q test revealed an absence of significant directional horizontal pleiotropy and heterogeneity, suggesting a high level of stability in the current MR study.

**Conclusions:**

According to our research, there is a direct causal link between increased circulating plasminogen levels and a higher risk of AD. Further studies are warranted to evaluate the underlying mechanism of our findings.